# Knowledge and decisions about maternal immunisation by pregnant women in Aotearoa New Zealand

**DOI:** 10.1186/s12913-022-08162-4

**Published:** 2022-06-14

**Authors:** Amber Young, Nadia A. Charania, Natalie Gauld, Pauline Norris, Nikki Turner, Esther Willing

**Affiliations:** 1 Division of Health Sciences , Kōhatu—Centre for Hauora Māori, Otago Medical School, University of Otago, PO Box 56 , 9054 Dunedin, New Zealand; 2 Department of Public Health, School of Public Health and Interdisciplinary Studies, Auckland University of Technology, Auckland, New Zealand; 3 Department of Paediatrics: Child and Youth Health, University of Auckland, Auckland, New Zealand; 4 School of Pharmacy, University of Auckland, Auckland, New Zealand; 5 Va’a o Tautai - Centre for Pacific Health, University of Otago, Dunedin, New Zealand; 6 Immunisation Advisory Centre, Department of General Practice and Primary Care, University of Auckland, Auckland, New Zealand

**Keywords:** Maternal immunisation, Maternal vaccination, Māori health, Pacific health, Health inequity, Informed choice

## Abstract

**Background:**

Maternal vaccinations for influenza and pertussis are recommended in New Zealand to protect mothers and their infant from infection. However, maternal immunisation coverage in New Zealand is suboptimal. Furthermore, there is unacceptable inequitable maternal immunisation rates across the country with Māori and Pacific women having significantly lower maternal immunisation rates than those of other New Zealanders.

**Methods:**

This research set out to explore what pregnant/recently pregnant Māori and Pacific women knew about immunisation during pregnancy and what factors influenced their decision to be vaccinated. A semi-structured interview guide was developed with questions focusing on knowledge of pertussis and influenza vaccination during pregnancy and decision-making. Māori and Pacific women aged over 16 years were purposively sampled and interviewed in Dunedin and Gisborne, New Zealand between May and August 2021. Interviews were analysed following a directed qualitative content approach. Data were arranged into coding nodes based on the study aims (deductive analysis) informed by previous literature and within these participant experiences were inductively coded into themes and subthemes.

**Results:**

Not all women were aware of maternal vaccine recommendations or they diseases they protected against. Many underestimated how dangerous influenza and pertussis could be and some were more concerned about potential harms of the vaccine. Furthermore, understanding potential harms of infection and protection provided by vaccination did not necessarily mean women would choose to be vaccinated. Those who decided to vaccinate felt well-informed, had vaccination recommended by their healthcare provider, and did so to protect their and their infant’s health. Those who decided against vaccination were concerned about safety of the vaccines, lacked the information they needed, were not offered the vaccine, or did not consider vaccination a priority.

**Conclusions:**

There is a lack of understanding about vaccine benefits and risks of vaccine-preventable diseases which can result in the reinforcement of negative influences such as the fear of side effects. Furthermore, if vaccine benefits are not understood, inaccessibility of vaccines and the precedence of other life priorities may prevent uptake. Being well-informed and supported to make positive decisions to vaccinate in pregnancy is likely to improve vaccine coverage in Māori and Pacific Island New Zealanders.

## Background

Vaccination is an effective global strategy to prevent or reduce morbidity and mortality caused by certain infectious diseases [1]. Vaccinations are recommended at different stages of life, including from birth (e.g. hepatitis B vaccine in infants exposed to the virus) right through to use in older adults (e.g. varicella-zoster) [2]. In many countries, including Aotearoa New Zealand, it is recommended that pregnant women are vaccinated for COVID-19, influenza, and pertussis to protect both themselves and their infant from infection [2–4].

Until recently, only two maternal vaccinations were recommended and provided free of charge in New Zealand, the pertussis-containing vaccine and the influenza vaccine [2, 4]. In 2021, the COVID-19 vaccine was also included in the maternal recommendations due to the risk of severe disease resulting from infection during pregnancy [2, 4]. The influenza and COVID-19 vaccines are recommended at any stage during pregnancy and the pertussis-containing vaccine (Tetanus-diphtheria and acellular pertussis) is recommended from the beginning of the second trimester [4].

Influenza infection during pregnancy can cause severe disease and hospitalisation [5], and poorer outcomes in infants (e.g. preterm birth, low birthweight, and hospitalisation) [4, 6]. In severe cases, the mother or her unborn baby may die [2]. Infants that are infected with pertussis (whooping cough) can become seriously ill and one in two infants infected will require hospitalisation [4]. Of those hospitalised, some suffer from severe morbidity such as brain damage and may die [7]. Influenza immunisation during pregnancy builds antibodies in the mother to protect them from infection. Additionally, following maternal immunisation against influenza and pertussis, antibodies are passed through the placenta to provide passive immunity to their infant during their first few months of life [2]. This protects infants from severe illness until they can be fully immunised themselves. In New Zealand, immunising pregnant women has been shown to reduce infection with influenza during pregnancy by approximately 50% and hospitalisation by 65% [2] and to prevent infant pertussis hospitalisations by 38% and deaths by 49% [8].

Maternal immunisation coverage in New Zealand is suboptimal [9]. Immunisation against pertussis infection during pregnancy was only taken up by 44% of pregnant women in 2018 [9]. Furthermore, Māori (Indigenous New Zealanders) and Pacific women and women from areas of high deprivation continue to have significantly lower maternal immunisation rates than those of other New Zealanders [9]. Māori and Pacific infants are also more likely to require hospitalisation with pertussis than those from other ethnic groups [10]. This is an outcome of unacceptable inequitable maternal immunisation rates with consequences across the country.

Maternity care in New Zealand is available free of charge as a national midwifery continuity of care model [11, 12]. This model allows time to build trusting partnership with women over time [12]. Trusting midwives recommendations is a known factor to support vaccine uptake [13]. However, the reasons that pregnant women remain unimmunised are often complex and multifactorial [14, 15]. Some women may not receive adequate information about recommended vaccinations during pregnancy [14], particularly if they live in areas of high deprivation [16], resulting in lack of awareness in these groups that vaccinations are recommended during pregnancy [14]. They may also be uncertain about the risks and benefits of vaccinating [17], possibly fuelled by misinformation [14]. Furthermore, access to health service providers is necessary to be immunised, yet barriers to accessing these health services may be significant for some [18–20]. Barriers to accessing midwifery care may be a particular concern for Māori and Pacific women [21–23].

## Methods

### Aims

Māori and Pacific women experience significant inequities in immunisation coverage during pregnancy [9]. This research set out to explore primarily what Māori and Pacific women knew about immunisation during pregnancy and what factors influenced their decision to be vaccinated to inform recommendations to maternal immunisation service delivery and reduce immunisation inequities.

### Study design and setting

Interviews were conducted in Dunedin and Gisborne, New Zealand between May and August 2021. Dunedin has a population of over 134,000, of which approximately 11% are Māori and 3% are Pacific peoples; Gisborne has a population of over 50,000, of which approximately 53% are Māori and 4.5% are Pacific peoples [24–26]. Interviews were held in a location that suited the women. In Dunedin they were conducted at a local community outreach centre, at a room booked at the University, and at women’s houses. In Gisborne, the interviews were conducted in a primary care health centre.

Tikanga Māori (Indigenous customary practices) were incorporated within the interview process and Community-Up research principles were practised [27, 28]. Specifically, Māori participants were offered a karakia (prayer or incantation) at the beginning of interviews, and researchers worked to build whanaungatanga (relationships). Manaakitanga (respect and hospitality) was promoted by providing participants with resources and support throughout the project, including a supermarket voucher as an appreciation of participation. For some of the interviews infants and small children were present, but for most interviews only the participants and researchers attended. Female Māori and Pacific research assistants supported the interviews (e.g. if translation was needed) and analysis of data for cultural context.

### Interview topic guide

A semi-structured interview guide was developed with questions focusing on knowledge of pertussis and influenza vaccination during pregnancy and decision-making. COVID-19 vaccination was not recommended in pregnancy until after some interviews had taken place, so COVID-19 vaccination was excluded from the topic guide. Concepts to be discussed during the interviews were determined by prior review of the literature and with input from the research advisory team, which consisted of Māori and non-Māori health research academics, a general practitioner, and pharmacists. The study protocol was peer reviewed independently by a Pacific health researcher and an academic public health physician and their feedback led to further improvement of questions for participants. The interview guide was then pilot tested with three Māori or Pacific women and no further amendments were made.

### Participants and recruitment

Participant number needed to be large enough to justify the appropriateness of themes identified from analysis and take into account limitations of time available and costs. We sought participation from 10 to 15 pregnant or recently pregnant women (defined as those whose infant was one year old or less). We undertook purposive sampling of Māori or Pacific women over 16 years of age, aiming to include those who received and did not receive maternal pertussis and/or influenza vaccinations. To be eligible for inclusion, participants needed be able to respond to questions in English. However, a Samoan research assistant provided translation support for two participants who needed additional help understanding the questions.

Recruitment was undertaken using a variety of methods including via posters in General Practice surgeries and in selected areas in the community, local midwives, posting on social media, by telephone through local community outreach and engagement, and snowballing. Written informed consent was obtained before each interview; this was done by reading through the consent documentation with the women, which included information about audio recording the interview. Two Samoan women needed additional support in understanding the English version of the consent documentation, so this was also read out verbatim in Samoan by the research assistant to ensure full understanding.

### Methodology and Analysis

The focus of this study is on pregnant women’s beliefs and decision-making around vaccination to inform recommendations to improve maternal immunisation service delivery. Thus, an interpretive description methodology was appropriate to provide a framework to discover patterns amongst women’s experiences [29].

The interviews were analysed following a directed qualitative content approach [30, 31]. The interviews were transcribed verbatim and double checked by the Primary Investigator (PI) and research assistants. As part of the immersion stage, the interview transcripts were reread multiple times by the PI. The PI, assisted by the Māori Investigator on the project, arranged data into coding nodes based on the study aims (deductive analysis) informed by previous literature and within these, data were inductively coded using qualitative analysis software programme NVivo Plus (QSR International LLC). Potential themes and sub-themes were reviewed against the dataset, and further refined by collapsing and reordering themes and data grouping into higher order themes for abstraction. Themes were then peer reviewed by the Māori Investigator on the project and reviewed by a participant for validation of the analysis; all reviewers agreed with the analysis and no changes were made.

The TACT framework (Trustworthiness, Auditability, Credibility, and Transferability) was used to enhance rigour [32]. Trustworthiness was achieved through clearly defining where data were collected from and through the collaboration with an experienced and diverse research team. Furthermore, a systematic process was utilised during analysis by organising data into the deductive nodes (i.e. the aims) and then constructing themes within these. Reflexivity is an important aspect of trustworthiness. The primary investigator (PI) is a woman who has been pregnant and a healthcare professional working in a Māori health research centre. The PI is supportive of maternal vaccinations and acknowledges how prior assumptions and behaviour may impact the study. The PI engaged in practices related to researcher reflexivity, such as identifying and acknowledging values and beliefs and how these may impact upon those with opposing views and keeping a researcher journal throughout the research process [33, 34]. Auditability was attained through clear description and transparency of data collection processes, verification of data transcriptions, and data analysis. Credibility was sought by sending themes to three participants for verification, peer review of analysis, and the use of direct quotations. Transferability was shown through clearly defining participants and the context of the study.

## Results

Interviews took approximately 20 min to one hour. Fifteen individuals participated in the project, nine identified as Māori, one identified as New Zealander/Māori, three identified as Samoan, and two as Cook Island Māori. Interviews in Gisborne led to more Māori women being recruited due to population demographics and working with a Māori health provider for recruitment. Ages ranged from 20 to 37 years of age. Seven were pregnant at the time of the interview and one of these also had a six-month-old infant. The remaining eight participants had infants aged one year or younger. Two participants (3 and 6) had limited English but were able to respond to questions with translation support from the Pacific research assistant (i.e. some questions required translation into Samoan). Eleven participants were born in New Zealand, three were born in Samoa and one was born in the Cook Islands. Deprivation was derived using participants street address and the Socioeconomic deprivation profile on the Environmental Health Intelligence New Zealand website; the lower the decile, the lower the level of deprivation [35]. The level of deprivation according to address was varied across the participants. Two lived in Decile 1–2 (area of lowest deprivation), three lived in Decile 3–4, one lived in Decile 5–6, two lived in Decile 7–8, and seven lived in Decile 9–10 (area of highest deprivation). See Table 1 for the ethnicity, timing of pregnancy or post-birth, and vaccination status of participants.

**Table 1:** Ethnicity, timing of pregnancy or post-birth, and vaccination status of participants

Participant number	Ethnicity	Stage of pregnancy/age of infant	Vaccination status
1	Cook Island Māori	Infant is 3 months old	Vaccinated against: - Influenza - Pertussis
2	Cook Island Māori	Infant is 6 months old	Not vaccinated
3	Samoan	Infant is 11 months old	Vaccinated against: - Influenza - Pertussis
4	Samoan	Infant is 2 months old	Vaccinated against: - Influenza
5	Māori	39 weeks pregnant	Vaccinated against: - Influenza
6	Samoan	Infant is 6 months old and currently 3 months pregnant	Not vaccinated
7	Māori	8 months pregnant	Vaccinated against: - Pertussis
8	Māori	Infant is 9 months old	Vaccinated against: - Influenza
9	Māori	34 weeks pregnant	Not vaccinated
10	Māori	Infant is 3 months old	Not vaccinated
11	Māori	Infant is 12 months old	Not vaccinated
12	New Zealander/ Māori	8 months pregnant	Not vaccinated
13	Māori	6 months pregnant	Will vaccinate against: - Pertussis
14	Māori	6 months pregnant	Undecided
15	Māori	Infant is 3 months old	Vaccinated against: - Pertussis

### Analysis

The nodes and themes identified from the interviews are shown in Table 2. Themes in the node Influences on decisions were split into Decided to vaccinate and Unvaccinated or undecided.

The main themes identified in this node are Gaps in awareness, Vaccination: Possible protection vs. potential harms, and Infection: Perceived potential harm and risk of infection. Little difference was found between Māori and Pacific participants across the themes.

**Table 2:** Themes and sub-themes identified

Node	Themes
**Knowledge about vaccination in pregnancy**	• Gaps in awareness • Vaccination: Possible protection versus potential harms • Infection: Perceived potential harm and risk of infection
**Influences on decisions**	**Decided to vaccinate**
	• Being well-informed is important • Clear recommendations have impact • Understanding protective benefits
	**Unvaccinated or undecided**
	• Previously held beliefs and practices • Need more effective discussions • Not prioritised

#### Gaps in awareness

Not all women were aware of maternal vaccine recommendations. Two participants were not aware of any vaccine that was recommended in pregnancy. Ten participants were aware of both vaccines, although when asked what they knew about the vaccines, some indicated they did not know much.

“I don’t really know a whole lot about the influenza immunisation, it’s kind of just there, it’s always been mentioned but it’s never been, like, talked about.” [P14]

“I think I didn’t know much and I still am not probably fully aware.” [P15].

A few participants were only aware of one of the recommended vaccines.

“Oh, I didn’t have that [the pertussis vaccine]. I didn’t quite have that info, just the flu one.” [P4]

#### Vaccination: Possible protection versus potential harms

Ten participants were aware that vaccines can protect against infection. Some thought protection was only for themselves, some thought it was only for their infant, and others thought protection was for themselves and their infant.

“It improves your health system, and it will also keep baby safe. Like, you know if it’s still in your womb... When they come out, they are still little and if sick you can’t do anything about it until they’re old. So, it’s easy for me to take the vaccination while baby in the womb so it helps keeps them safe as well.” [P4]

“…if I got vaccinated, while pregnant, the baby would have lots of immunity in her own self.” [P15]

Although, having a small amount of knowledge that protection from infection occurred, did not incentivise some women to vaccinate.

“I don’t really know much about them. Just that they protect you from those things, yeah… would just protect you and your baby.” [P11]

Some participants’ vaccination perceptions centred around potential harms. Safety concerns heard from family and friends were closely linked to influences on vaccination, as described under Unvaccinated or undecided. None of these participants were vaccinated against pertussis during pregnancy and only one [P8 quoted below] was vaccinated against influenza because it was recommended by her employer.

A third of the participants were concerned about the safety of vaccines; however, this was often a vague recollection of an adverse event discussed by friends or family.

“Their baby has ended up in Starship [New Zealand paediatric hospital] or something and they claimed it is from a vaccine… within my circle of friends. Um yeah that scares you a little bit… because it is so personalised.” [P8]

“My cousin, actually, she’s in a wheelchair from being immunised, but that wasn’t while her mum was pregnant. I think it was after. But that whole thing there has put me off altogether. I get my kids done… But not while I am pregnant.” [P9]

Two unvaccinated participants had been discouraged by their peers’ comments about safety of vaccines.

“Yes, I did hear information from other women you know they tell me “Don’t take it because if you take it you’re gonna get sick… side effects, this is what is going to happen to you”.” [P2]

#### Infection: Perceived potential harm and risk of infection

The perceptions of vaccine preventable disease varied amongst participants. Some women were not aware of the diseases at all. Some were aware that diseases could be harmful if contracted, but many underestimated how dangerous these diseases could be for the mother or their infant. Most women felt at risk of catching influenza, but not of pertussis. However, even knowing potential harms of pertussis and influenza and the potential risk of catching these infections did not necessarily mean women would choose to be vaccinated. Some believed that the diseases could be prevented by other means, your body could fight infection sufficiently, or could be treated if infection was contracted.

Not all participants understood the potential harms of pertussis infection and a few participants had not heard about pertussis infection in early infancy. Over half of the women interviewed knew that contracting pertussis could be harmful to infants. Many had a vague notion of the disease being ‘bad’ or ‘serious’ in infants but were not aware that it can be fatal.

“I just know that if babies do get it [pertussis], it can be really bad… for baby it would be awful… but just from what I looked up I just know that it can be really, I don’t know if it can be fatal or not.” [P15]

A third of participants did not know much about pertussis or underestimated the potential harms in infants.

“I don’t know [how serious is it] because I haven’t, I don’t know anything about it.” [P10].

“…it’s another sickness that, you know, they’ve got to go through.” [P5].

Just over half of the participants acknowledged, to varying degrees, that influenza infection could be a problem for the pregnant mother, their infant, or for both of them.

“Really serious, like really bad. It [influenza] would be a concern for me and especially baby.” [P4]

However, almost all (*n* = 13) participants either underestimated the potential harms of influenza infection or did not know infection could be worse in pregnancy, for some this was because they had experienced influenza before and it had been mild. Nine of these participants did not receive the influenza vaccination.

“I didn’t actually think it would be that bad to be quite honest.” [P8]

“I’ve always just been told it can’t harm the baby.” [P7]

Knowing potential seriousness of influenza or pertussis infection did not always influence women’s vaccine decision making. Some participants who remained unvaccinated also thought it would be serious for them and/or their infant.

“I think it [influenza] would be serious, not only for me but also for my baby, yeah... yeah could definitely be concerned about it, it could come to the worst of it as well.” [P2]

“[Pertussis infection would be] serious for the baby I think, yeah… what I’ve seen, like, on social media that babies have less of a chance of surviving.” [P11]

The likelihood of catching influenza and pertussis was discussed. Overall, participants thought it was more likely they could catch influenza than pertussis. Most participants thought the likelihood of catching influenza was high. However, almost half were not vaccinated even though they felt they would be susceptible to infection.

“Quite [likely to get influenza], I get quite sick quite easily.” [P5].

Four participants thought the likelihood of catching influenza was low and were not vaccinated. Reasons of perceived low risk included the introduced COVID-19 mitigation strategies and infant’s due date being at the end of the influenza season.

“You know with, like, COVID and everything now, everyone’s a lot more, you know, signs their name, washes their hands, sanitises, some people even wear masks and things like that.” [P12]

“Not very likely [to catch influenza]… just because its coming to the end of winter.” [P13].

Over half of the participants did not think it was likely they or their infant would catch pertussis and only a couple of these women were vaccinated against it. For many, this was because they had not been personally affected by pertussis before.

“I guess I thought it [pertussis infection] was not very likely.” [P8]

“While growing up it was not common where I come from. Whooping cough, I never heard of it. I’ve never have heard of any case, growing up in the Cook Islands, that any babies passed away from that kind of thing.” [P2]

A group of participants who did not want the vaccine while pregnant thought alternative measures could be taken to prevent infection or harm from disease if contracted. These participants thought you could take precautions to prevent infection (e.g. frequent handwashing and keeping warm), or they would prefer to rely on their immune system to counter infection, or they thought the best course of action would be to seek medical help once they had been infected.

“I think I need to be careful and make myself comfortable and also dress up warm… yeah I think that’s enough.” [P6]

“I feel your body can help itself if it needs to.” [P13].

#### Influences on decisions

The second node in the framework is about the influences on participants’ decisions about whether to vaccinate during pregnancy. This node had many themes, grouped into two context topics of Decided to vaccinate and Unvaccinated or undecided. Grouping enabled the influences to be put into context of the outcome (i.e. whether they were vaccinated). Firstly, for Decided to vaccinate, there were three main themes: Being well-informed is important, Clear recommendations have impact, and Understanding protective benefits. Secondly, for those who were Unvaccinated or undecided, there were also three main themes: Previously held beliefs and practices, Need more effective discussions, and Not prioritised. For many, their reasons for vaccinating or not vaccinating were down to a multitude of factors (i.e. they appear in more than one theme or subtheme).

##### Decided to vaccinate

Many participants decided to vaccinate because they felt informed enough to make that decision, usually by their midwife or sometimes by their General Practitioner (GP; i.e. their family doctor). For one, being well-informed changed their decision to be vaccinated. Some participants decided to vaccinate because it was recommended by their midwife or GP, or because influenza vaccination was offered in their workplace. Some participants were encouraged to vaccinate by their family and friends. Some participants decided to vaccinate to protect their health and the health of their infant. The themes are summarised in Table 3.

**Table 3:** Positive influences on decision to vaccinate

Theme (n)	Comment and example quotes
Being well-informed is important (*n* = 6)	For some participants, being adequately informed about vaccinations was enough to decide to be vaccinated. “…I read all the information, also my doctor explain[ed] to me what this means. And I’m OK. I’m ok to do it.” [P3] “I wasn’t going to, but when I got more information on it [from the midwife], I decided it would be better for my baby… I actually wasn’t going to get it and then I decided that I should.” [P7]
Clear recommendations have impact (*n* = 8)	A third of participants decided to get vaccinated because it was recommended by their GP or midwife. Three participants were positively influenced by their family or friends. A couple of the participants were recommended and offered influenza vaccination through their place of work. “Midwife they tell me to do that thing [get vaccinated].” [P3] “It was my partner’s aunty actually [that told her about vaccination]. So, I just booked an appointment with the doctor.” [P5] “I did have the flu vaccine though. That’s only because we are offered it through work. But otherwise out of work, I don’t know.” [P8]
Understanding protective benefits (*n* = 11)	Seven participants decided to vaccinate to ensure their baby was healthy. Four participants wanted to get vaccinated for their own health and safety. “I decided it would be better for my baby… the health of my baby.” [P7] “For baby, and also for the mum as well.” [P3]

##### Unvaccinated or undecided

Some participants did not want to vaccinate because of previously held beliefs or practices. Some were concerned about safety of the vaccines and thought the risks did not outweigh potential benefits. Two participants distrusted their healthcare provider’s opinion on the topic, with one believing that healthcare providers follow the Ministry of Health recommendations rather than give independent advice that was best for their clients. The other had limited contact with their midwife and preferred to rely on family for healthcare matters. Concern about vaccine safety and distrust limited participants’ ability to receive positive information about vaccinating during pregnancy. Many participants thought they were unlikely to catch influenza or pertussis. For some, because the perceived risk of contracting the disease was low, they did not think they needed to be vaccinated. Some participants had chosen to not get vaccinated because they do not usually get vaccinated, or they had not been vaccinated for earlier pregnancies so did not think it was necessary.

Several participants remained unvaccinated because they lacked the information they needed. For example, some were not aware of the vaccines and not offered them by their healthcare provider. Others were not given sufficient information to make them comfortable with being vaccinated, e.g. an adequate amount of safety information. Five participants heard information from family and friends that made them decide against vaccination and these concerns were often not discussed with their midwife or GP. Other times, participants were aware of vaccines because of a previous pregnancy but because they had not been offered them for their most recent pregnancy, they thought they were not needed. Three participants were aware of vaccination but did not consider this a priority during their pregnancy and the reasons that vaccination was not prioritised differed. Two participants were busy with work and hadn’t been offered one or both vaccines so had not followed up because they did not think it was important; both had limited knowledge on the potential severity of infection in them or their infant. The other participant was concerned about vaccine safety and did not make it a priority to find out more information or to seek further advice. The themes are summarised in Table 4.

**Table 4:** Negative influences on decision to vaccinate

Theme	Comment and example quotes
Previously held beliefs and practices (*n* = 9)	Some did not get vaccinated because of concerns around the safety of the vaccine for them or their unborn baby. “I don’t want to get anything while I am pregnant. Anything at all, because it may have effects. That’s what I am scared of.” [P9] “I’m sure that there’s a lot of, it’s just a worry that if I do take this like what could potentially go wrong with it.” [P14]
	Some did not trust the advice on vaccination from their healthcare provider. “I’d trust what they’re saying, but I still like to go and do some research because I’m not interested in vaccinating… we could all just go ‘oh yes’ and you know do everything like a robot sort of thing but then there’s also choices… Health professionals are just doing what the government says and how the Ministry of Health are doing things whereas, like, if you wanted to find alternatives and other things like that, you’d have to go and find it yourself.” [P12] “Not really [trust healthcare provider advice about vaccination]… just my beliefs… women have die[d] following vaccination.” [P6]
	Some decided to not vaccinate because of perceived low likelihood of catching the disease. “It wasn’t [a consideration] at the time, just because I don’t normally get the flu.” [P15] “Not concerned because none of my kids have ever had it.” [P10]
	Some did not think vaccination was necessary because they do not usually get vaccinated or had not been vaccinated for previous pregnancies “I chose not to get the flu vaccine while I was pregnant and that was just because I’ve never gotten the flu vaccine before so I kind of just figured, just to keep going as normal.” [P15] “I didn’t have it with my first one, so I don’t know why I had to have it with my second one.” [P8]
Need more effective discussions (*n* = 11)	Many participants felt they were not given enough information to decide to vaccinate. Some were not offered the vaccine, and some were unaware of vaccine recommendations. “Just that lack of understanding, what actually is that… It’s just that lack of [information], because all that you are given is that brochure, yes, I’ll read it but I’m the type that would like to know more… It’s not just what’s on the brochure, I need to know more.” [P2] “I didn’t have the whooping cough [vaccine]. That’s because it wasn’t offered to me.” [P8]
	Some described having doubts after discussing vaccination with family and friends. “I didn’t want to get it because I have heard that if you get it, it actually brings it [influenza] on.” [P9] “Other people have their doubts and then they start putting stuff in your head and you’re like, “should I trust this?”.” [P11]
Not prioritised (*n* = 3)	Some were aware of recommendations, but vaccination was not a priority during their pregnancy. “I just made excuses because I forgot or didn’t just have time. But it was never a priority for me to make that decision.” [P2] “I just haven’t got round to it, because I only just finished work two weeks ago.” [P5]

## Discussion

This study aimed to explore what Māori and Pacific women knew about immunisation during pregnancy and what factors influenced their decision to be vaccinated. Fifteen participants were interviewed, five identified as Pacific Island ethnicity and ten as Māori. A 2008 Canadian study demonstrated that high levels of knowledge and positive attitudes towards maternal influenza vaccination and recommendations from health care providers were associated with maternal vaccination [36]. A 2013 review, incorporating studies undertaken from 1997 to 2012 in ten different countries, also established clear recommendations and removal of barriers to vaccination assisted with influenza vaccine uptake [37]. More recent New Zealand studies also found similar factors supported immunisation uptake such as health professional recommendations and free administration [14, 15, 17]. Like these other studies, our study identified that being well informed and receiving both clear recommendations, prioritisation, and support for ease of access for vaccination in pregnancy underpins a positive decision-making process. However, most women in this study were not adequately supported by health services to decide to vaccinate during pregnancy. For example, they were not offered a vaccine by their healthcare providers, they were not given sufficient or appropriate information about vaccination as a priority, they were unaware of the potential harm from vaccine-preventable diseases, or they were misinformed about the safety of vaccines. These negative influences on vaccine decision-making were also reported in international [37] and other New Zealand studies [14, 15, 17]. Improving understanding about the importance of vaccination in pregnancy has long been understood to be vital for successful vaccination coverage [17, 37] and could help overcome negative perceptions, hesitancies, indifferent attitudes and low prioritisation to improve coverage in marginalised groups.

Knowledge about pertussis and influenza infection is often limited [15, 38, 39] and this was demonstrated in this study with most participants underestimating the potential harm from influenza for themselves and pertussis in their infant. Perceiving influenza as harmless is a common misconception [37] and this study showed this is still a widely-held belief. Furthermore, some participants had never heard of pertussis or whooping cough before and although many others thought pertussis could be somewhat serious in infancy, the severity of infection was often not entirely understood. This differed to another New Zealand study where most participants (*n* = 441, 74%) had received vaccination [17]. However, in this other study more than half of the women were aware of a currently active pertussis pandemic and many had encouraging information from their midwife (*n* = 327, 54.9%) and/or their GP (*n* = 226, 37.9%). Furthermore, there was an underrepresentation of Māori (4.5%) and Pacific women (1.6%) compared to the general population (8.4% and 2.6% respectively at the time of the study) [17]. It has been shown that a predictor of vaccination acceptance was awareness of the benefits of being vaccinated [37, 40] and knowing the potential harm from disease [40]. However, some women in this study who acknowledged the potential for harm from influenza and pertussis and the possibility of contracting these diseases still chose not to vaccinate. This indicates they may not truly understand the severity of these infections or attribute the risk of disease in themselves and their infant. This lack of knowledge and understanding of pertussis and influenza contrasted with a higher concern of vaccine safety and potential harms amongst participants. The concern about vaccine safety is a commonly held concern and is an often-stated reason of avoiding maternal vaccination. Besides these concerns, as described elsewhere [17, 37], many participants were unaware of the availability one or both recommended vaccines. To encourage maternal vaccination in Māori and Pacific women in New Zealand, individuals need to understand benefits of vaccinating, the potential harm from diseases, and the likelihood of infection. However, it is important that these points are discussed in relation to perceived vaccination harms, addressing concerns pregnant women may have. Health care professionals need to not simply mention the vaccinations but provide information in a way that women can understand and prioritise because perceptions of vaccines and vaccine-preventable diseases influence the decision to vaccinate during pregnancy [40]. Face-to-face education and resources for parents that focus on awareness of disease, particularly for protection for infants, needs to be prioritised.

New Zealand [15] and international studies [41] have shown that some women do not receive influenza vaccination because they believed that because they were ‘healthy’ the vaccination was not necessary [15]. Similarly, in our study two participants thought their body could fight influenza infection so did not need vaccination as a prevention measure. Two other participants who did not get vaccinated thought they could rely on other means to prevent infection. This included keeping warm in winter and infection prevention measures learned through the COVID-19 pandemic [41]. Other participants would rather seek help and get treatment if they were infected, as seen in other studies [37]. In view of the severity of influenza and pertussis [4], when these beliefs are used as a replacement for immunisation or reduces the prioritisation of maternal immunisation then this is a concern. This issue needs to be considered within the education materials and conversations.

There were many influences that impacted decisions to vaccinate. One commonly found influence in this study and others is the desire to protect themselves and/or their infant from infectious diseases [14, 17, 42]. Also, in this study, being adequately informed about vaccination had a clear impact. In another New Zealand study with a high proportion of vaccinated participants [17] midwives were singled out as providers of the most helpful advice and encouragement for vaccinations in pregnancy. Furthermore, strong recommendations to vaccinate, particularly by healthcare providers were positively associated with decisions to vaccinate rather than a passive provision of information, and again, other studies have also supported this [14, 17, 37, 38].

Individuals who decide not to vaccinate or were still undecided are often influenced by their perception of low likelihood of disease [14, 37], because they do not usually get vaccinated [19, 37], or because they do not consider it a priority [43]. Interviews with Māori women and Māori health providers about maternal vaccinations found the health providers often noting women not prioritising vaccinations [15]. The current paper has provided greater insight from women as to why they do not prioritise, and the lack of information they often had. Women in our study also expressed these influences can be addressed with support from healthcare providers. Helping women understand the risk of disease will encourage them to make vaccination a priority whilst they are pregnant, and this could be achieved through effective and empathetic discussions [15]. As mentioned above, education with a focus on awareness of disease is important to accomplish this.

Participants also display hesitancy towards vaccination due to concern over safety of vaccines and these concerns must be discussed with openness and understanding to support informed decision-making. Family and friends can have a profound influence on vaccine uptake, and it is known that when vaccination is strongly supported by family and friends, uptake is increased [39, 40]. However, discouraging information can often be promulgated by family and friends [39]. In contrast to other studies about maternal vaccination [15], women in this study were strongly influenced by their support network, and most of the time this influence prevented them from being vaccinated. Sometimes this was a general recommendation about not knowing if the vaccine is safe and other times this was from accounts of family members adversely affected by historical vaccination. Once again, this demonstrates the need for open communication to discuss concerns about vaccination and also supports the need for wider education to family and other members of mothers’ support networks.

There are a number of barriers to vaccination for pregnant women to overcome that have been described in New Zealand and abroad including lack of transport, lack of available time due to other commitments (e.g. childcare, work), and difficulty in accessing appropriate services [15, 38, 40]. Removing barriers and making access to vaccinations easy for pregnant women is recommended to improve vaccination rates in pregnancy [15]. Furthermore, it is widely recognised that educating mothers and improving rapport and discussions with healthcare providers are important strategies to improve vaccine uptake during pregnancy [37, 44] and some unvaccinated participants in this study identified that they were not well-supported to make positive decisions. However, not knowing about vaccination is a common reason why women remain unvaccinated during pregnancy [14, 19, 20, 41]. In this study, seven participants reported not being offered maternal vaccinations and so were unaware of them being available or the lack of discussion made them think vaccination was not important. Additionally, two participants did not trust advice provided by their healthcare professional and distrust in recommendations has been described as a factor in women not receiving maternal influenza vaccine internationally [45]. Furthermore, distrust in recommendations combined with limited knowledge of the severity of vaccine-preventable disease would ‘compound the problem’ [46]. Conversely, it has been identified that trusting a healthcare professional improves perceptions of vaccine efficacy and safety [37]. Trust has been identified, along with effective health promotion and public engagement, as part of a multiple system strategy required to protect New Zealand from future measles epidemics and this should be also pulled to the forefront for increasing maternal immunisation coverage [47].

This research was novel because it focused on maternal vaccination knowledge and influences on vaccine uptake in Māori and Pacific women. Many findings were in line with previous research, but this study also found that Māori and Pacific women may be negatively influenced by their family and friends. This emphasises the need to allow time to build trusting relationships with pregnant women and set aside time to discuss women’s concerns with them in a safe environment. Trusting relationships is essential so women are comfortable sharing their fears and open to the information provided by their healthcare provider. Furthermore, this research showed that being able to describe harms of vaccine-preventable disease and the potential for infection did not mean that women would choose vaccination. When discussing vaccination with women, it is important to determine they are aware of the importance of vaccination as it relates to them and their infant. Further research is needed to determine how women assess the benefits and risks of vaccination when they can discuss the harms but still choose to not vaccinate.

### Strengths and limitations

A strength of this study is the purposive sampling of Māori and Pacific women to help give a better insight into why these groups may not be vaccinating. Other New Zealand studies investigating maternal immunisations had Māori or Pacific groups under-represented [15, 17]. Twelve participants had not received one or both maternal vaccinations, providing useful data on potential influences on decision-making for such women. Also, the interviews were reasonably long in duration to allow the topic to be discussed in-depth.

This study had a small sample size from only two locations in New Zealand. However, small sample sizes are common with qualitative research [48] and a range of views were expressed. Although we interviewed a broad range of ages, we did not interview women under 20 years old. Women in this group may be less likely to be immunised [9, 49], so their opinions should be sought to gain insight into the perceptions of this group. Although, twelve of the participants in our study remained unvaccinated or only singly vaccinated, there may be some similarities that could overlap with a younger cohort. Attempts were made to gather feedback from participants for validation of the themes identified. Six participants were approached and three agreed to review. Only one participant read through the themes and replied so certainty could not be obtained. The participant who did reply, felt the themes were appropriate and reflected the sentiment of her comments.

Despite these limitations, this study is supported by findings from other New Zealand and international studies. The fact that there is crossover from findings from studies from over a decade ago [36, 37] indicate that further strategies are required for effective change to improve maternal vaccination rates, particularly for Māori or Pacific women who have inequitable maternal vaccination coverage. Recommendations to improve vaccination coverage have ranged from additional funded visits to GPs and nurses to discuss immunisations [17], training of health care professionals [50], funded availability through pharmacies [51], administration in the antenatal visit setting, constant vaccine reminders throughout pregnancy [18, 43], large public health campaigns, and removal of barriers to access [42]. Ultimately, a multi-pronged method to enhance vaccination uptake is needed, with improving knowledge of vaccination in pregnancy underpinning the approach.

## Conclusions

Understanding that vaccines are safe to be given during pregnancy and why they are recommended is essential, but these elements may be unknown, misunderstood, or considered irrelevant by some Māori and Pacific women. Information provided in New Zealand may not always be sufficient or clear enough to improve knowledge and help individuals to make an informed decision. Furthermore, there may be a lack of strong recommendations to vaccination by healthcare professionals to raise priority for action. The lack of understanding about vaccine benefits and risks of vaccine-preventable diseases, alongside lack of ease of accessibility, can result in the reinforcement of other negative influences such as the fear of side effects or the precedence of other life priorities. Being well-informed and supported to make positive decisions to vaccinate in pregnancy is likely to improve vaccine coverage in Māori and Pacific Island New Zealanders.

## Data Availability

Data sharing is not applicable to this article as no datasets were generated or analysed during the current study.

## Declarations

All participants gave written informed consent for the study prior to undertaking the interviews. All participants were asked and consented to the interviews being audio recorded.
